# ZnO nanoparticles inhibit the activity of *Porphyromonas gingivalis* and *Actinomyces naeslundii* and promote the mineralization of the cementum

**DOI:** 10.1186/s12903-019-0780-y

**Published:** 2019-05-14

**Authors:** Jingyu Wang, Lele Du, Yingmei Fu, Peidong Jiang, Xiumei Wang

**Affiliations:** 1 Department of Dentistry, the Second Affiliated Hospital of Harbin Medical University, Harbin, 150086 China; 2 Wu Lien-Teh Institute, Department of Microbiology, Harbin Medical University, The Heilongjiang Key laboratory of immunity and infection, Pathogen Biology, Harbin, 150081 China

**Keywords:** Zinc oxide nanoparticles, Antimicrobial, Apical periodontitis, Dental material, Cytotoxicity

## Abstract

**Background:**

Zinc oxide nanoparticles (ZnONPs) have been widely studied as bactericidal reagents. However, it is still challenging to use ZnONPs as a root canal sealant to eliminate infecting microorganisms in the root canal system. This study aimed at understanding the antibacterial and biofilm effects of ZnONPs in the infected root canal and their effect on cell function.

**Methods:**

This study aimed to develop a better understanding of the antibacterial effects of ZnONPs in the infected root canal and their effect on cell function. Experiments were performed in two stages; the first stage included inhibition zone tests and the minimum inhibitory concentration (MIC) test, which were performed to examine the antibacterial activity of ZnONPs against *Porphyromonas gingivalis* (*P. gingivalis*) and *Actinomyces Naeslundii* (*A. naeslundii*) bacteria in vitro. ZnONPs were further evaluated for their biocompatibility using normal mouse NIH3T3 and OCCM-30 cells by the cell-based MTT assay. In addition, the influence of ZnONPs on matrix metalloproteinases in NIH3T3 cells and their inhibiting factors (Mmp13 and Timp1) were measured using the real-time PCR technique and western blot method.

**Results:**

The MIC of ZnONPs against *P. gingivalis* and *A. naeslundii* were confirmed to be 10 μg/mL and 40 μg/mL, respectively. The MTT assay showed that ZnONPs were nontoxic. The RT-PCR and western blotting results showed that Mmp13 was downregulated and Timp1 expression was increased. Meanwhile, ZnONPs were shown to increase the expression of the OCCM-30 osteogenesis-related factors Bsp and Runx2. Finally, there was no significant change in the morphology of NIH3T3 and OCCM-30 cells after the addition of different concentrations of ZnONPs for different periods of time.

**Conclusion:**

ZnONPs have excellent antibacterial activity against *P. gingivalis* and *A. naeslundii* and have low cell cytotoxicity in vitro.

## Background

Dentistry involves the treatment of several oral diseases, of which apical periodontitis is a major disorder that affects the majority of the population. Bacteria are the main cause of apical periodontitis and cause apical inflammation and bone destruction. Therefore, the purpose of root canal therapy is to eliminate bacteria from the root canal system and prevent future infections [1]. In endodontic treatments, mechanical instrumentation combined with antibacterial irrigation effectively reduces the rate of bacterial infection [2]. However, the elimination of microorganisms is challenging due to the complex morphology of the root canal system, which includes various dentin tubule morphologies, apical tube branching, isthmus, and irregularities. It is challenging to achieve a sterile root canal system and eliminate infected debris [3]. Root canal treatment often fails due to bacterial infection after root canal filling. Thus, fillers with long-lasting antimicrobial properties may be the preferred choice for more durable root canal filling materials.

Nanomaterials have become increasingly common for biomedical applications and have shown great potential as new drugs to kill or inhibit numerous microorganisms [4, 5]. Benefiting from their excellent antibacterial properties and low cytotoxicity, a few nanomaterials have been successfully applied in many fields, including nanomedicines, antibacterial surfaces, protective clothing, food preservation, water treatment, and disinfecting agents [6, 7]. In recent decades, many researchers have reported the antibacterial activity of Zinc oxide nanoparticles (ZnONPs) on different microorganisms, such as *Escherichia coli* [8], *Campylobacter jejuni* [9], *Pseudomonas aeruginosa* [10] and *Vibrio cholerae* [11]. At present, nanotechnology is used to produce a large number of dental materials, including light-cured restorative composite resins and their bonding systems, impression materials, dental implant covering layers and fluoride mouthwashes [12, 13]. However, reports of ZnONPs as root canal filling materials with an antibacterial effect are still rare. Furthermore, the potential toxicity of nanomaterials has aroused attention in recent years [14, 15]. Nanotoxicology studies performed directly on cultured cells have provided significant information regarding the effects that nanomaterials might have on humans and other species [16].

In this manuscript, we characterized ZnONPs with a size of 10 nm by transmission electron microscopy (TEM) and optimized the concentration of ZnONPs with powerful antibacterial activity according to the minimum inhibitory concentration (MIC) and minimum bactericidal concentration (MBC) against *Porphyromonas gingivalis* (*P. gingivalis*) and *Actinomyces Naeslundii* (*A. naeslundii*)*.* We added different concentrations (0 μg/mL, 1/8 MIC, 1/4 MIC, 1/2 MIC, or MIC) of ZnONPs to draw antibacterial curves against *P. gingivalis* and *A. naeslundii.* In addition, the effects of ZnONPs on the cell morphology and proliferation of NIH3T3 and OCCM-30 cells at different time points and concentrations were determined. More importantly, the influence of ZnONPs on these two cell types was evaluated. For this purpose, we examined the expression of Mmp13 and Timp1 in NIH3T3 cells and the expression of Bsp and Runx2 in OCCM-30 cells after ZnONPs were added.

## Methods

### Bacterial culture

*P. gingivalis* (ATCC33277) was obtained from the Central Laboratory of Capital Medical University (Beijing, China). Bacteria were routinely grown in BHI broth (BBL Microbiology Systems, Cockeysville, MA, USA) supplemented with 0.001% hemin and 0.0001% vitamin K (THB-HK) and sterile sheep blood. *A. naeslundii* (ATCC19246) was obtained from the West China School of Stomatology Sichuan University (Chengdu, China). BHI broth was used to determine the viable growth of microbes from their freeze-dried form. All strains were anaerobically cultured at 37 °C for 48 h. The turbidity of the two strains in a test tube confirmed the growth of microbes, which was compared and adjusted to a 0.5 McFarland turbidity standard (10^8^ colony forming U/mL).

### Cells and cell culture conditions

The cementoblast cell line (OCCM-30) was kindly provided by Dr. Hongchen Sun (School of Stomatology, Jilin University); normal mice fibroblast cells (NIH3T3) were stored in the Laboratory of Medical Genetics, Harbin Medical University. NIH3T3 cells were cultured in Dulbecco’s Modified Eagle’s Medium (DMEM, Lonza, Walkersville, MD, USA). OCCM-30 cells were cultured in F12 medium (Lonza). All cells were supplemented with 10% (v/v) foetal bovine serum (FBS) (PAA Laboratories GmbH, Pasching, Australia) and 1% penicillin/streptomycin (GIBCO, Germany) (100 U/mL penicillin and 100 μg/mL streptomycin) according to the supplier’s protocol and were cultured at 37 °C in a humidified 5% CO_2_ atmosphere.

### Preparation of ZnONPs

ZnONPs were obtained from the Technical Institute of Physics and Chemistry, CAS (Beijing, China). Zinc salt (0.015 M, Aldrich) and 0.1 g of dimethyl sulfone (Aldrich) were first dissolved in 80 mL methanol (Beijing Chemical Works) with continuous stirring at 60 °C. Subsequently, 40 mL of 0.001 M KOH (Aldrich) was added at a rate of 1 mL per minute, and the reaction was kept at 60 °C for 12 h to obtain 10 nm ZnONPs. The precipitate was washed three times with ethanol to remove soluble impurities, dried at 65 °C for 12 h, and stored at room temperature before use.

### Transmission electron microscopy

The size and morphology of the ZnONPs were examined using a transmission electron microscope.

### Determining the MIC of ZnONPs

The Gram-negative bacteria *P. gingivalis* and *A. naeslundii* were used to study the antibacterial activity of ZnONPs. All apparatuses and materials were autoclaved and handled under sterile conditions during the experiments. A sterile 96-well plate was used in the MIC test. The MIC of uncoated ZnONPs was evaluated using the broth dilution method. Using a wide concentration range of ZnONPs, between 1.25 and 320 μg/mL, the double dilution test was performed at a selected concentration. The density of the bacteria in the liquid cultures was estimated by optical density (OD) measurements at a wavelength of 600 nm. The bacterial suspension used to determine antibacterial activity contained 1 × 10^6^ colony-forming units (CFU) mL^− 1^.

### Determining the antibacterial curve of ZnONPs

*A. naeslundii* and *P. gingivalis* were incubated in BHI broth with ZnONPs. The experimental concentrations (v/v) of ZnONPs for *A. naeslundii* were 0 μg/mL, 5 μg/mL, 10 μg/mL, 20 μg/mL, and 40 μg/mL. *P. gingivalis* cultures were also treated with different concentrations (0, 1.25, 2.5, 5, or 10 μg/mL) of ZnONPs. The concentration of both strains was 10^6^ CFU/mL; cells were seeded into 96-well plates and cultured under anaerobic conditions at 37 °C for 48 h. To each well, 100 μL of ZnONPs and 100 μL of bacterial suspension were added. The antibacterial kinetic curves were created based on the absorbance of the optical density at 600 nm (OD_600_) as determined by a spectrophotometer. Two separate experiments were carried out in triplicate.

### Agar diffusion test

The agar diffusion test was performed under strict aseptic conditions in a class II, type A2 biological safety cabinet. The antimicrobial activity of the materials was evaluated by the method of agar diffusion. A base layer of 10 mL of BHI agar was poured on Petri plates (90 × 15 mm). After the solidification of the agar, a second layer of 10 mL of BHI agar supplemented with 0.001% hemin and 0.0001% vitamin K (THB-HK) and sterile sheep blood was poured on the first layer for the cultivation of *P. gingivalis*. The second layer of *A. naeslundii* was still BHI agar. After solidification of the second layer, all four sealers were placed in the wells at a depth of 4 mm and were punched at a diameter of 6 mm in 20 plates of BHI agar. The surface of each agar plate was inoculated by swabbing with 0.5 McFarland turbidity standard suspensions of *P. gingivalis* and *A. naeslundii*, which applied evenly with a glass rod on 10 Petri plates per bacteria. ZnONPs, AH-Plus (Dentsply Sirona, Pennsylvania, USA), root filling agent (Shanghai Eryi Zhangjiang Biomaterial Co, Shanghai, China) and sterile distilled water were prepared according to the manufacturer’s instruction. Preparations of the AH-Plus and root filling agent included equal proportions at 1:1 in a sterilized glass plate, and the sealer was carefully placed in the wells with the aid of a sterilized spatula. These plates containing *P. gingivalis* were cultured under anaerobic culturing conditions at 37 °C for 5~7 d. *A. naeslundii* was cultured for 24~48 h as described above. The diameters of the zones of inhibition around each well were measured in millimetres (mm) of incubation. The mean diameter of the measured zone was determined for all four sealers.

### Cell viability assay

The cytotoxicity of ZnONPs was determined by the 3-(4,5-dimethylthiazol-2-yl)-2,5-diphenyltetrazolium bromide (MTT) viability assay. OCCM-30 and NIH3T3 cells were seeded at a density of 2 × 10^3^ cells/well in 96-well microplates. Cells were treated with different concentrations of ZnONPs (0, 2.5, 5, 10, 20 or 40 μg/mL) for 5 d. The cells with added ZnONPs were measured once every 24 h in a series of doses, and 20 μL fresh MTT solution was added to each well for 3~4 h. The viability rate (%) was calculated from the relative absorbance at 600 nm with respect to the percentage of the control. The percentage of cell viability was calculated as MTT cell activity, % = (Aexp −Aneg)/(Acon–Aneg), where Aexp is the experimental group absorbance, Aneg is the blank group absorbance, and Acon is the control group absorbance. The data of three independent experiments were analysed with two-way *ANOVA*.

### Cellular morphology assay using an inverted microscope

Cells were seeded into 6-well plates and treated with different concentrations (0, 1.25, 2.5, 5, or 10 μg/mL) of ZnONPs. Images of the NIH3T3 and OCCM-30 cell morphology were taken at 0, 12, 24 and 48 h. The experiments were performed in triplicate.

### Real-time PCR analysis

Total RNA was isolated and transcribed into complementary DNA using the Transcriptor First Strand cDNA Synthesis Kit (Roche, Basel, Switzerland). Real-time PCR was performed using a LightCycler®480 fluorescence quantitative PCR instrument. The primers for Mmp13 mRNA and other factors in mice were as follows: Mmp13 (forward): 5′-TGT TTG CAG AGC ACT ACT TGA A-3′; Mmp13 (reverse): 5′-CAG TCA CCT CTA AGC CAA AGA AA-3′; Timp1 (forward): 5′-CGA GAC CAC CTT ATA CCA GCG-3′; Timp1 (reverse): 5′-ATG ACT GGG GTG TAG GCG TA-3′; Runx2 (forward): 5′-TTC AAC GAT CTG AGA TTT GTG GG-3′; Runx2 (reverse): 5′-GGA TGA GGA ATG CGC CCT A-3′; Bsp (forward): 5′-ATG GAG ACG GCG ATA GTT CC-3′; Bsp (reverse): 5′-CTA GCT GTT ACA CCC GAG AGT-3′; β-actin (forward): 5′-AAA TCT GGC ACC ACA CCT TC-3′; β-actin (reverse): 5′-GGG GTG TTG AAG GTC TCA A-3′.The data of three independent experiments were analysed with two-way *ANOVA*.

### Western blot analysis

Cells at the logarithmic phase were harvested and lysed with radioimmunoprecipitation assay (RIPA) buffer [150 mM NaCl, 1% NP-40, 0.25% Na-deoxycholate, 1 mM ethylenediaminetetraacetic acid (EDTA), 50 mM Tris-HCl, pH 7.4]. Total protein and nuclear proteins were extracted. Proteins were separated by 10% sodium dodecyl sulphate polyacrylamide gel electrophoresis (SDS-PAGE) and transferred onto polyvinylidene difluoride (PVDF) membranes (Millipore, Billerica, MA, USA). The membranes were incubated with a primary antibody overnight at 4 °C, followed by a secondary antibody (Zhongshan Bio-Tech Co, Beijing, China) for 1 h at room temperature. Then, the membranes were scanned using an Odyssey infrared imaging system (LICOR, Lincoln, NE, USA). The primary antibodies against Mmp13, Timp1, Bsp, Runx2 (Proteintech Group, Inc., Wuhan, China), and GADPH (KangChen Biotech, Shanghai, China) were used for western blot analysis.

### Statistical analysis

The data are expressed as the mean ± SD. Statistical analyses included t-tests and *ANOVA* using the GraphPad software. Statistical significance was determined at *P* < 0.05.

## Results

### TEM analysis of ZnONPs

**Fig. 1:**
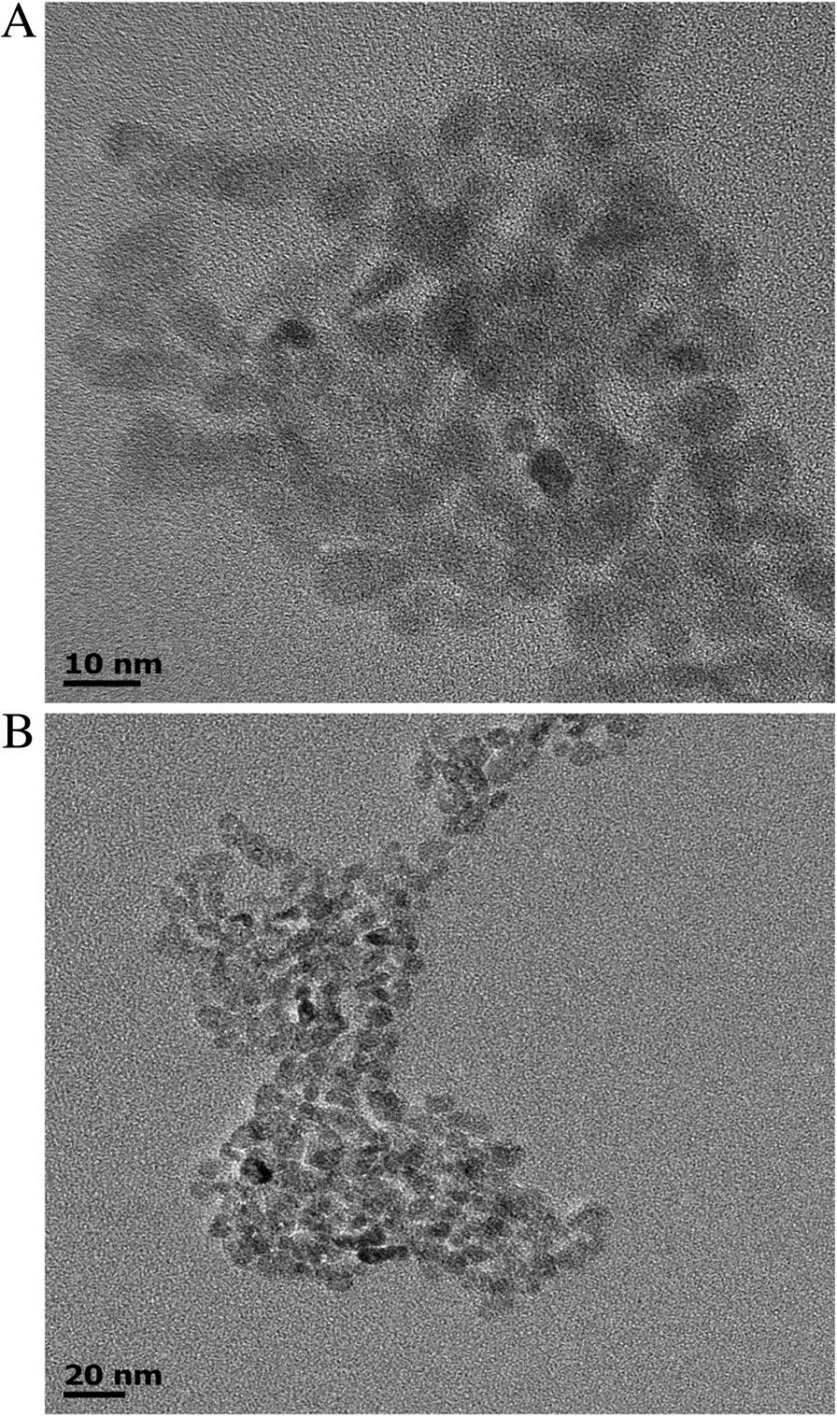
**a**, **b** TEM images of 10 nm ZnO nanoparticles under different magnifications

The size and shape of the ZnONPs were characterized from the TEM images shown in Fig. 1a and b. The ZnONPs were found to be approximately 10 nm in diameter, and they were almost spherical and uniformly dispersed.

### ZnONPs had bactericidal effects against *A. naeslundii* and *P. gingivalis*

The antibacterial effects of ZnONPs against *A. naeslundii* and *P. gingivalis* were verified by two established MIC and MBC tests. The broth dilution method determined the MIC of ZnONPs against *A. naeslundii* and *P. gingivalis*, 40 μg/mL and 10 μg/mL, respectively. However, the bactericidal activity determined by the MBC was more relevant than the bactericidal activity determined by the MIC for the evaluation of the antimicrobial activity in dental composites. The bacterial solution of the 96-well plate was cultured on BHI agar, and the minimum concentration without bacterial growth was MBC. The MBC for *A. naeslundii* and *P. gingivalis* were 80 μg/mL and 40 μg/mL, respectively.

### Bactericidal curve of ZnONPs on *A. naeslundii* and *P. gingivalis*

**Fig. 2:**
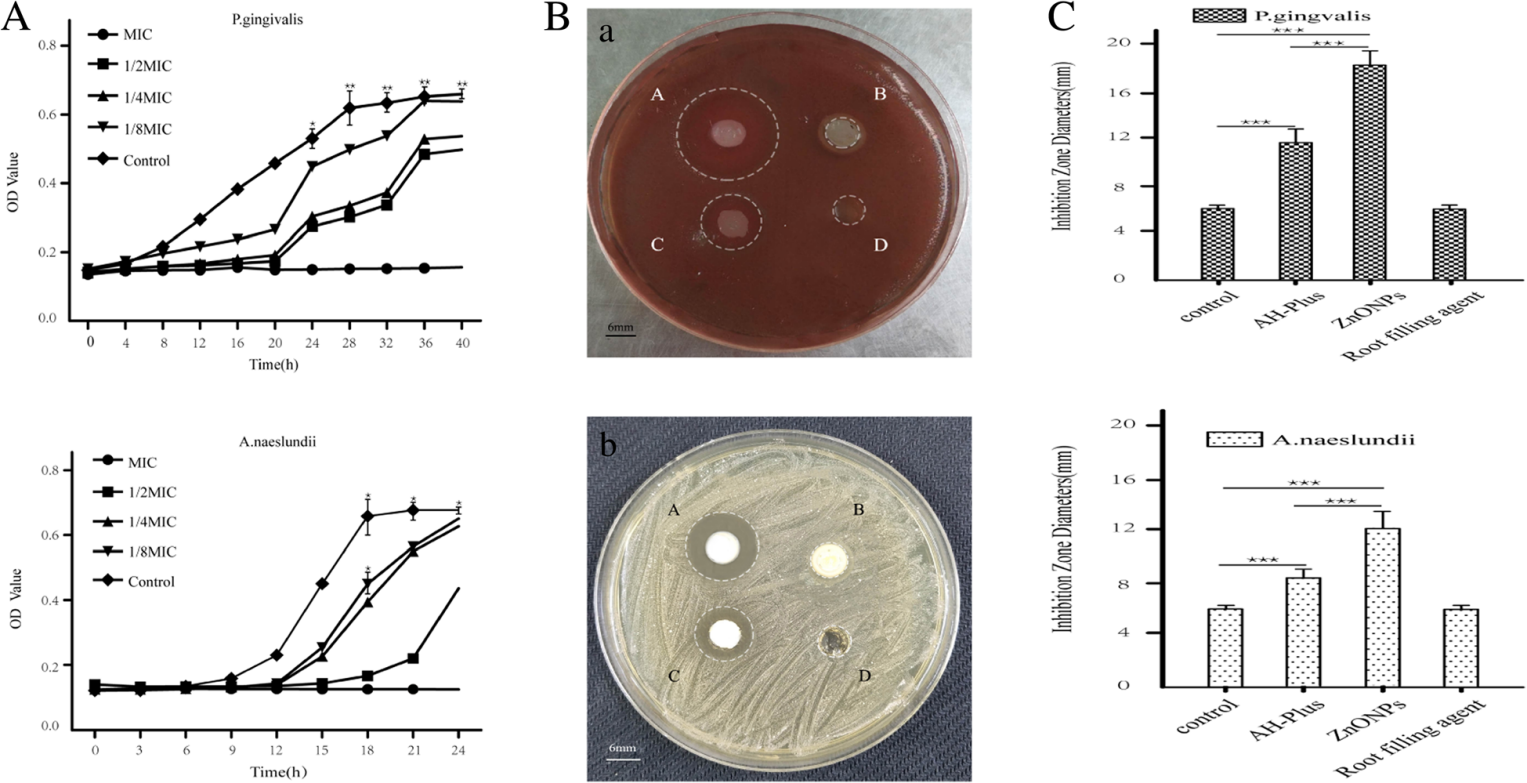
Results of antimicrobial curve tests and inhibition zone tests of ZnONPs at different concentrations. **a** Effect of ZnONPs on the growth curves of *P. gingivalis* (a) and *A. naeslundii* (b)*.* **b** Images of the inhibition zone of *P. gingivalis* (a) and *A. naeslundii* (b) with different materials. (A~D) ZnONPs, root filling agent, AH-Plus and control groups, respectively. **c** Diameters of the inhibition zone of different materials against *P. gingivalis* (a) and *A. naeslundii* (b). The data are shown as the mean ± SD of the diameters of the inhibition zone. *Indicates *P* < 0.05, **indicates *P* < 0.01, ***indicates *P* < 0.001 by *ANOVA*

The antibacterial curves of ZnONPs against *A. naeslundii* and *P. gingivalis* are shown in Fig. 2a (a, b). It can be seen that the growth rate of *A. naeslundii* and *P. gingivalis* significantly slowed after the addition of 1/2 MIC, 1/4 MIC and 1/8 MIC of ZnONPs and that the logarithmic period and stationary phase were postponed. ZnONPs inhibited the growth of *P. gingivalis* and delayed logarithmic growth at 12 h for a long time; the inhibition effect ended at 36 h. At 12 h, *A. naeslundii* entered the logarithmic growth period, but the stable growth period was postponed to 24 h. At the MIC of ZnONPs, the OD value of the bacteria suspension did not change with time and bacteria growth stopped. The inhibitory effect of ZnONPs on *A. naeslundii* and *P. gingivalis* was apparent.

### ZnONPs have superior antibacterial activity compared to AH-Plus and the root filling agent

To further investigate the difference between the ZnONPs and the root canal sealer, antibacterial activity and agar diffusion tests were conducted in *P. gingivalis* and *A. naeslundii*. Figure 2b shows the results of the inhibition zone test of three different materials for the two kinds of bacteria. Figures A~D represent ZnONPs, the root filling agent, AH-Plus and control group, respectively. The results of the inhibition zones tests show reduced bacterial activity by measuring the area of the circular clear zones on the opaque background of bacterial growth. As shown in Fig. 2b, the bacteriostatic area of ZnONPs and AH-Plus were clear and there was a significant increase of ZnONPs antibacterial activity, but the root filling agent could not inhibit the growth of the bacteria. The diameters of the inhibition zones were measured with a Vernier calliper, as shown in Fig. 2c. The maximum inhibitory radii of ZnONPs on *P. gingivalis* and *A .naeslundii* were 18.09 mm and 12.05 mm, respectively. These results showed that ZnONPs on *P. gingivalis* and *A. naeslundii* had greater antibacterial activity than commercial AH-Plus and root filling agents.

### ZnONPs does not affect the cell proliferation of NIH3T3 and OCCM-30 cells

**Fig. 3:**
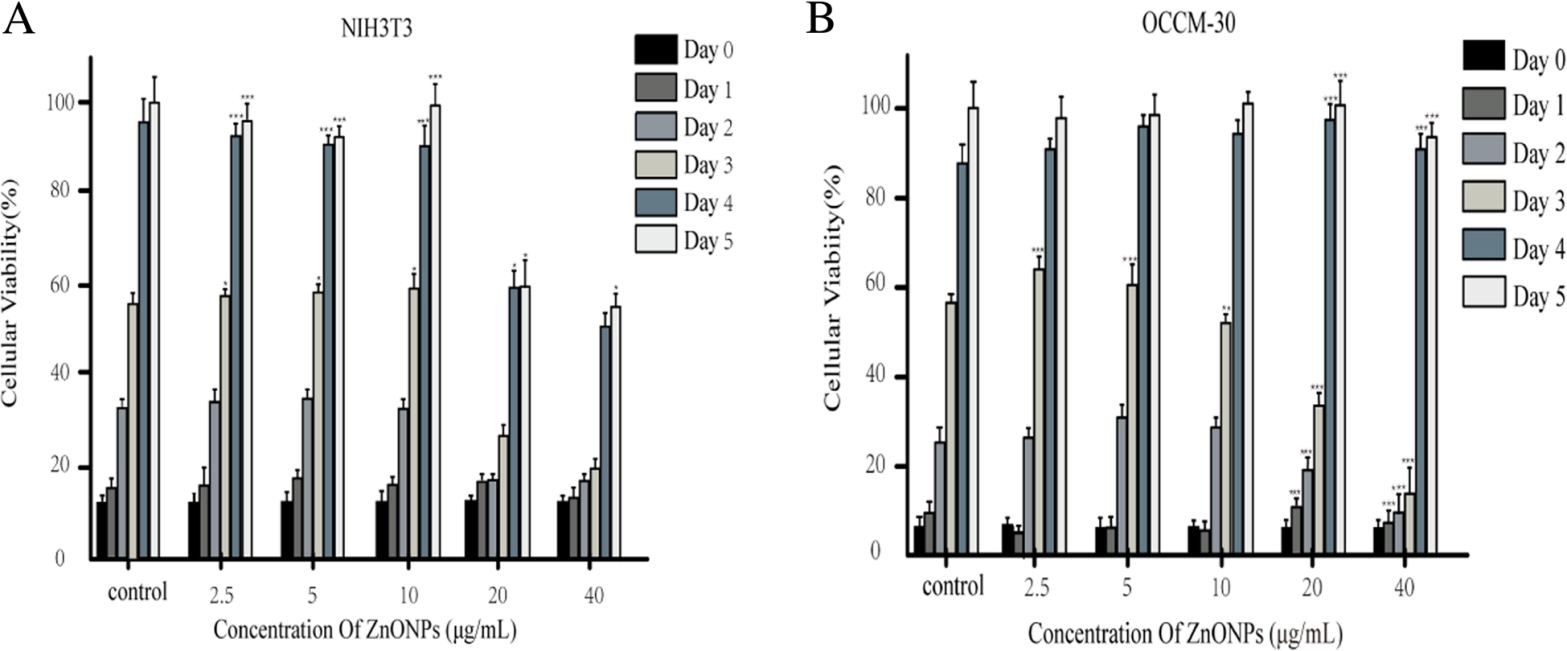
Effect of ZnONPs on NIH3T3 (**a**) and OCCM-30 (**b**) cells by the MTT assay. The percentage of absorbance at different concentrations of ZnONPs compared with that of the control was calculated. The OD value of the cells was measured every day for 5 d and plotted for the mean ± SD. **indicates *P* < 0.01, ***indicates *P* < 0.001 by *ANOVA*

For the cytotoxicity study, NIH3T3 and OCCM-30 cells were used. The viability of NIH3T3 cells exposed to ZnONPs for 5 d were all above 60% when the concentrations were 20 μg/mL and 40 μg/mL (Fig. 3a). When the concentration was 40 μg/mL, the survival rate of OCCM-30 cells exposed to ZnONPs for 5 d was not different from that of the control group (Fig. 3b), but cell proliferation was slow in the first three days. When the concentration was 10 μg/mL and below, within 5 d, ZnONPs did not affect the growth of the two kinds of cells. The IC50 values of ZnONPs on NIH3T3 and OCCM-30 cells were determined to be 43.45 μg/mL and 45.34 μg/mL, respectively. The MTT assay results indicate that ZnONPs showed no cytotoxicity.

### ZnONPs do not affect the cell morphology of NIH3T3 and OCCM-30 cells in vitro

**Fig. 4:**
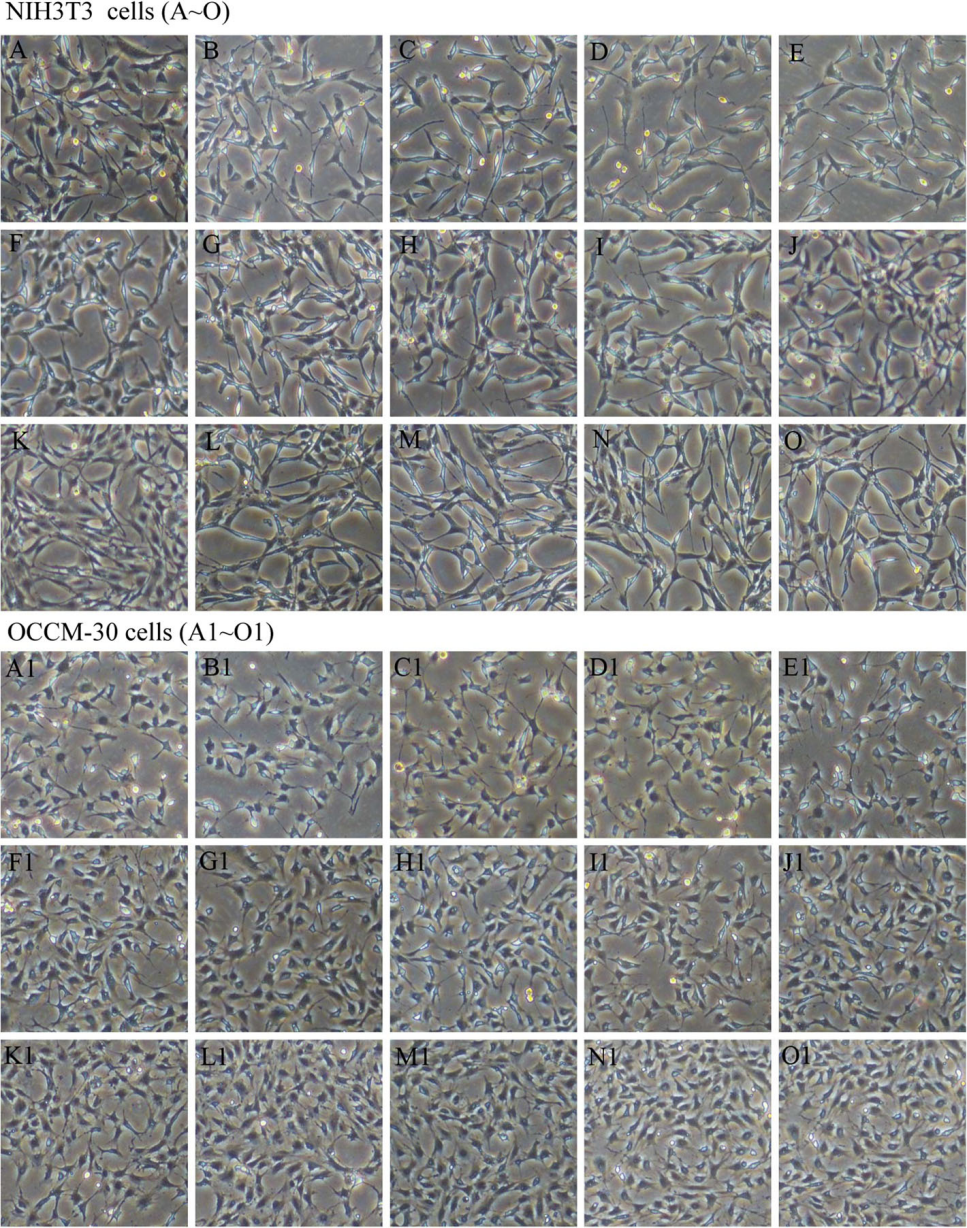
Cell morphology changes after adding ZnONPs. **a**-**o** Morphological appearance of NIH3T3 cells under a microscope. **a**, **f** and **k** Control cells; (**b**-**e**, **g**-**j** and **l**-**o**) cells treated with ZnONPs at concentrations of 2.5, 5, 7.5 and 10 μg/mL for 12 h, 24 h and 48 h. **a1**-**o1** show the morphological appearance of OCCM-30 cells under the microscope. ( a1, f1 and k1) Control cells; (**b1**-**e1**, **g1**-**j1** and **l1**-**o1**) cells treated with ZnONPs at concentrations of 2.5, 5, 7.5 and 10 μg/mL for 12 h, 24 h and 48 h (original magnification: 400x)

After NIH3T3 and OCCM-30 cells were incubated with 0, 2.5, 5, 7.5 and 10 μg/mL ZnONPs for 12, 24 and 48 h, the morphology of the cells was observed under a microscope (Fig. 4). Compared to the untreated control group, evident morphological changes were not seen in treated cells.

### ZnONPs decreased the expression of Mmp13 in NIH3T3 cells

**Fig. 5:**
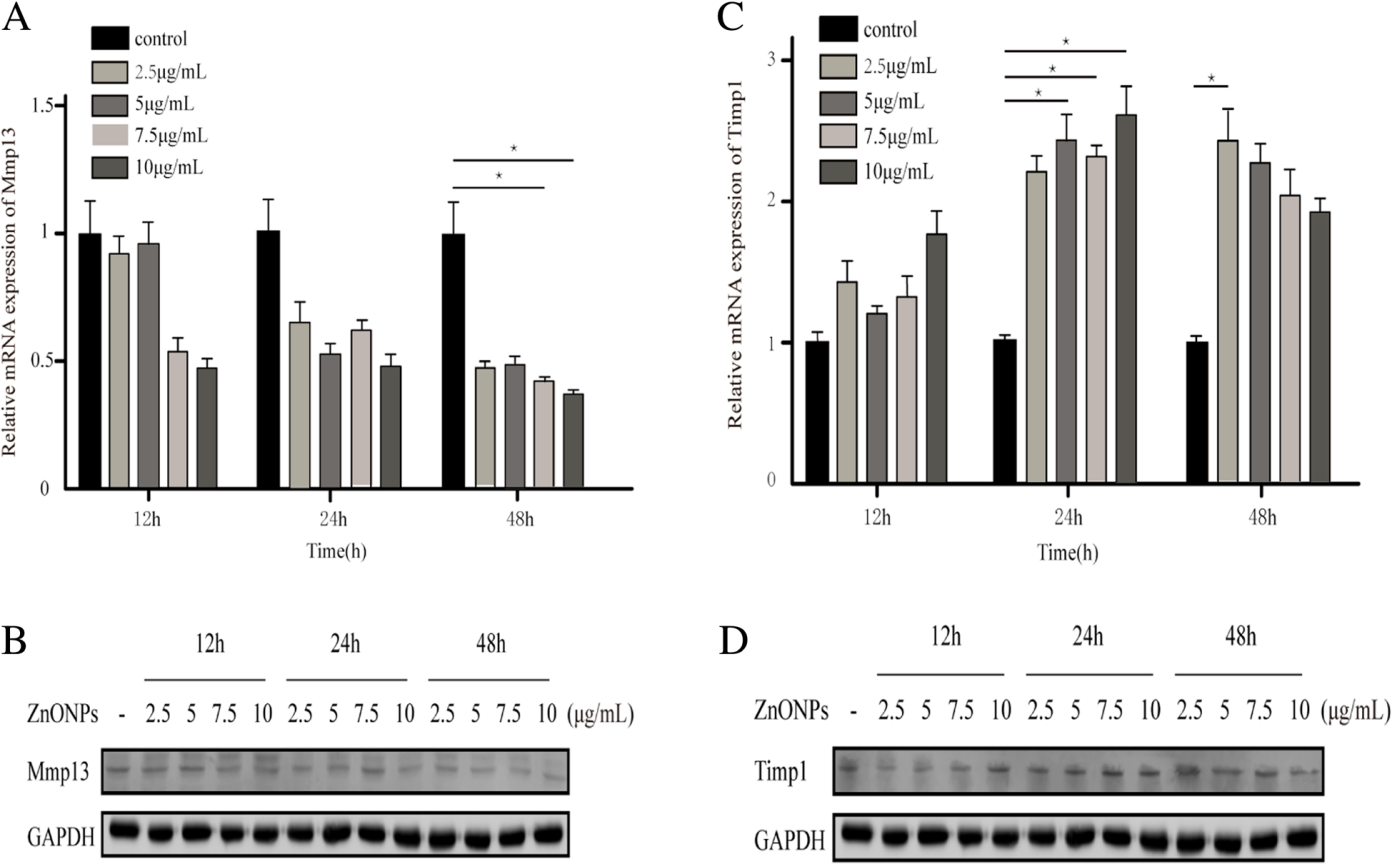
Expression of Mmp13 and Timp1 in NIH3T3 Cells after ZnONPs treatment. **a**, **b**: ZnONPs decreased Mmp13 mRNA and protein expression. **c**, **d**: ZnONPs increased Timp1 mRNA and protein expression. β-actin and GAPDH were detected as controls. *Indicates *P* < 0.05 by *ANOVA*. (SD, standard deviation)

To further evaluate the regulatory role of ZnONPs in NIH3T3 cells, the expression of Mmp13 was detected by RT-PCR and immunoblotting analyses. Compared with the control group, the expression of Mmp13 was gradually downregulated with the increase of the drug concentration and incubation time after the addition of ZnONPs; the lowest expression was at 48 h at 10 μg/mL (*P* < 0.05) (Fig. 5a and b).

In addition, we determined the Timp1 expression level in NIH3T3 cells. We found there was a positive correlation between Timp1 expression and ZnONPs and that its expression increased with the increase of the drug concentration and time; the expression was the highest at 24 h, and the difference was statistically significant compared with that of the control group (*P* < 0.05) (Fig. 5c and d). Taken together, these results indicate that ZnONPs can reduce the expression of Mmp13 in NIH3T3 cells and may inhibit tooth root absorption.

### ZnONPs induce the upregulation of the Bsp and Runx2 proteins in OCCM-30 cells

**Fig. 6:**
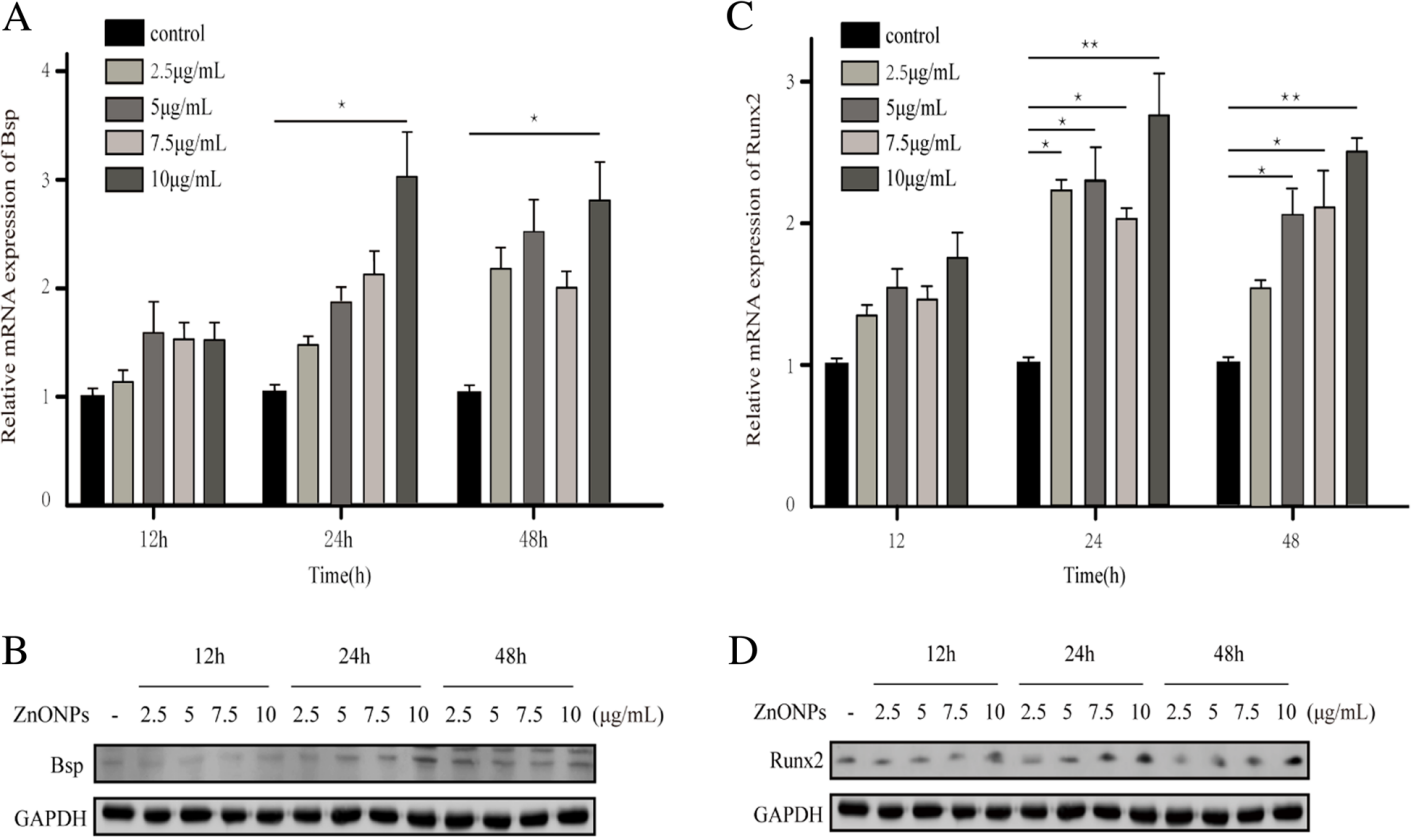
ZnONPs promoted the expression of Bsp and Runx2 in OCCM-30 cells. ZnONPs increased Bsp (**a**, **b**) and Runx2 (**c**, **d**) mRNA and protein expression. β-actin and GAPDH were detected as controls.*Indicates *P* < 0.05, **indicates *P* < 0.01. (SD, standard deviation)

To further study the effect of ZnONPs on cells, we performed the following analysis of ZnONPs. We used RT-PCR and western blotting to assess whether ZnONPs affected the expression of the osteogenic proteins Bsp and Runx2 in OCCM-30 cells. When incubating OCCM-30 cells with 0, 2.5, 5, 7.5 and 10 μg/mL ZnONPs for 12 h, 24 h, and 48 h, we found that the expression of Bsp and Runx2 increased compared to that of the negative control group and that the proteins were induced in dose- and time-dependent manners (Fig. 6). We also found that the expression of Bsp and Runx2 in 10 μg/mL ZnONPs was highest at 24 h and that the difference was statistically significant compared with that of the control group (*P* < 0.05). Therefore, ZnONPs were able to induce the upregulation of Bsp and Runx2 expression in OCCM-30 cells. Taken together, these data indicate that ZnONPs can increase the mineralization function of OCCM-30 cells and may affect the process of root resorption and repair.

## Discussion

Apical periodontitis should be considered an inflammatory reaction in periapical tissues to the presence of bacteria within the root canal system. In view of the particularity of the infected root canal environment and the complexity of bacteria, the pathogenesis of the clinical symptoms of apical periodontitis is still not very clear. As the main pathogenic factor of apical periodontitis, bacteria play an important role in the pathogenesis of the clinical symptoms of periapical periodontitis, including endotoxins, capsules, outer membrane proteins, proteolytic enzymes and their metabolites. Lipopolysaccharide (LPS), a cell wall component of bacteria, can induce osteoblasts to secrete inflammatory factors, such as IL-1β and IL-6. These cytokines can act on proosteoclasts and activate them into osteoclasts to play an osteolytic role, leading to inflammatory absorption and destruction of the periapical bone tissue. Endodontic bacteria fall into eight bacterial phyla, namely, Firmicutes, Bacteroidetes, Spirochaetes, Fusobacteria, Actino-bacteria, Proteobacteria, Synergistes, and TM7, of which the detection rates of *P. gingivalis* and *A. naeslundii* are high [17]. Studies have shown that Porphyromonas, Actinomyces, and others are associated with pain, swelling and sinus tracts in the root tip of teeth. Among bacteria, *P. melanogenica* and *P. gingivalis* are most closely related to acute periapical inflammation and malodour in root canals. Therefore, we take the elimination of pathogenic bacteria in infected root canals as the starting point, with the hope of preparing new drugs that can effectively reduce the virulence of LPS to control periapical inflammation symptoms and prevent bone destruction.

Characterization of ZnONPs was performed by TEM, which showed that their sizes were in the range of 1~100 nm in diameter. Since ZnONPs have small crystal grains, their surface electrons and crystal structure were changed, making ZnONPs highly transparent and highly dispersive [18]. As a new nanomaterial with excellent antibacterial properties and low cytotoxicity, application of ZnONPs in the field of medicine has gradually increased. In the present study, we tested the antibacterial effects of ZnONPs on the apical periodontitis pathogenic bacteria *P. gingivalis* and *A. naeslundii.* The growth of bacteria was inhibited at the MIC; therefore, the MIC of *P. gingivalis* (10 μg/mL ZnONPs) and *A. naeslundii* (40 μg/mL ZnONPs) were used as the maximum concentrations, and the concentration gradient of ZnONPs was set by two dilutions to prepare an antibacterial curve. We found that the inhibition rate of ZnONPs on *P. gingivalis* and *A. naeslundii* at 1/2 MIC was 80% and that the growth of bacteria was inhibited when the MIC was used. Satisfactory root canal filling materials should not only have a good sealing ability but also have the advantages of excellent cytocompatibility, tissue tolerance, and low solubility. If the root canal filling material has antimicrobial activity, it will help eliminate residual microorganisms that survive in chemical drugs and mechanical instruments, thus improving the success rate of the root canal treatment. Many studies have been performed to evaluate the antimicrobial activity of different endodontic sealers, and the most frequently used method to evaluate this activity is diffusion in agar. Nevertheless, this method does not only depend on the toxicity of the material against one microorganism but it can be influenced by the diffusion and affinity of the substance in the culture media. A material with easier diffusion will, consequently, produce greater inhibition halos. Therefore, in this study, we compared AH-Plus, a root filling agent and ZnONPs with similar fluidity. The results showed ZnONPs had a stronger antibacterial activity compared with that of the dental filling materials AH-Plus and root filling agent. Moreover, as an root canal sealer, the addition of ZnONPs can improve the sealing ability [19]. This enhanced antibacterial property is desirable in endodontic sealers because sealers can achieve good control of microbial infections by killing microorganisms in the dentinal tubules that come in contact and avoid reinfection. In summary, hydrophilic ZnONPs with small sizes and good monodispersity were prepared by a simple, green, sustainable, and low-temperature solvothermal method, and the produced ZnONPs showed excellent antibacterial properties at a low concentration. The detailed mechanism for the antibacterial activity of ZnONPs is still under debate. A possible mechanism can be explained on the basis of the oxygen species released on the surface of ZnO, which cause fatal damage to microorganisms. When ZnO with defects is activated by UV or visible light, electron-hole pairs (e^−^h^+^) can be created. The holes split H_2_O molecules into OH^−^ and H^+^. Then, dissolved oxygen molecules are transformed into superoxide radical anions and react with H^+^ to generate (HO_2_^•^) radicals, which upon subsequent collision with electrons, produce hydrogen peroxide anions (HO_2_^−^). Hydrogen peroxide anions then react with hydrogen ions to produce molecules of H_2_O_2_. The production of H_2_O_2_ can penetrate the cell membrane and achieve bactericidal action. There are other theories, including zinc ion release, mechanical damage of the cell membrane or cell wall, and the changes in pH value caused by ZnO in the reaction system [20]. In summary, the bactericidal properties of ZnONPs are complicated and involve multifactorial antibacterial mechanisms.

The increased application of nanoparticles in many fields suggests that a full and fundamental understanding of their potential toxicity is needed. The low cytotoxicity of ZnONPs in human cells is the fundamental requirement for their use as antibacterial agents. Various cell lines (normal diploid or transformed) are commonly used in cytotoxicity evaluations. Since cementoblasts and fibroblasts are critical to healthy periapical tissue, they were used in this study. No obvious morphological changes were seen in NIH3T3 and OCCM-30 cells exposed to lower concentrations of ZnONPs. There was no significant change in cell morphology after treatment with 10 μg/mL ZnONPs for 48 h. In the present study, the cytotoxicity of ZnONPs was evaluated by the MTT assay. It was found that the viability of NIH3T3 cells exposed to ZnONPs for 5 d was above 60% with 40 μg/mL ZnONPs. At the same concentration, ZnONPs delayed the division of OCCM-30 cells but did not affect the final cell proliferation. The MTT results indicated that ZnONPs showed low cytotoxicity. Enhanced toxicity of ZnONPs after increasing the concentration could be attributed to their high solubility in the extracellular region, which will, in turn, increase the intracellular Zn^2+^ levels, or may be due to their direct entry into the cell, which causes an increase of intracellular Zn^2+^ levels [21].

To further investigate the effect of ZnONPs on periapical tissue, we studied the effect of ZnONPs on NIH3T3 and OCCM-30 cells. Mmp13 belongs to the subfamily of collagen. During the degradation of the bone matrix, Mmp13 is secreted by osteoblasts and macrophages and initiates natural collagen I, II and III [22], which play pivotal roles in bone remodelling activation and pathological bone resorption [23]. Mmp13 is recognized as a potential target for bone resorption related diseases, such as osteoarthritis and periapical periodontitis [24, 25]. Our study examined the expression of Mmp13 and its inhibitor Timp1 at different time points with different concentrations of ZnONPs in NIH3T3 cells. RT-PCR and western blotting showed that the expression of Mmp13 decreased in a time- and dosage-dependent manner. By contrast, Timp1 increased with the increase of the drug concentration and time. These results support the hypothesis that ZnONPs inhibit Mmp13 secretion. Based on the above findings, ZnONPs may reduce periapical bone resorption by reducing the expression of Mmp13. Moreover, we also studied the expression levels of mRNA and proteins related to osteoblasts in OCCM-30 cells after the addition of ZnONPs. Bsp is a non-collagen that is highly expressed in the early stages of the formation of cementum and plays a very important role [26]. Its main function is to control the formation and development of the apatite crystal nucleus. In the process of cementum mineralization, the faster the formation of cementum, the greater the Bsp content detected. The interaction between Bsp and SPP1 plays an irreplaceable role in the formation of cementum [27]. Runx2, a specific transcription factor of osteoblasts, plays an important role in osteoblast differentiation and bone tissue formation [28]. Studies have shown that Runx2 can control the rate of bone formation by acting on the differentiated osteoblasts [29]. In this study, after the action of ZnONPs on OCCM-30 cells, the levels of Bsp and Runx2 mRNA in each concentration group significantly increased and protein expression was also significantly higher than that in the control group. We infer that ZnONPs can promote the differentiation and mineralization of cementoblasts by regulating the expression of Bsp and Runx2, which are related to osteoblasts.

## Conclusion

In summary, we found that ZnONPs can be used as a new alternative root canal filling material. In bacteriostasis, low concentrations of ZnONPs can inhibit the growth of bacteria, control the formation of biofilm, and eliminate pathogenic bacteria. It is helpful to determine the antibacterial effect of ZnONPs on bacteria in the infected root canal. The cytotoxicity experiment showed that the prepared ZnONPs had lower toxicity in vitro and had no effect on cell differentiation and morphology. Further, we found that ZnONPs can reduce the expression of Mmp13 related to bone resorption and also promote the expression of osteoblast-related proteins. These studies will provide very useful information for the future development of ZnONPs in the field of biomedicine. ZnONPs have broad biomedical application prospects in root canal disinfection.

## Abbreviations

*A. naeslundii*: *Actinomyces Naeslundii*
ATCC: American Type Culture Collection
BHI: Brain Heart Infusion
CFU: Colony Forming Units
MBC: Minimum bactericidal concentration
MIC: Minimum inhibitory concentration
*P. gingivalis*: *Porphyromonas gingivalis*
TEM: Transmission electron microscopy
ZnONPs: Zinc oxide nanoparticles
